# Five-flavor *Sophora flavescens* enteric-coated capsules for ulcerative colitis: ethnopharmacological rationale, pharmacology, clinical evidence, and safety

**DOI:** 10.3389/fphar.2026.1972684

**Published:** 2026-10-22

**Authors:** Chunyu Ma, Cuiping Yang

**Affiliations:** 1 The First Clinical Medical College, Bengbu Medical University, Bengbu, China; 2 Department of Gastroenterology, Ruijin Hospital, Shanghai Jiao Tong University School of Medicine, Shanghai, China

**Keywords:** ethnopharmacology, five-flavor *Sophora flavescens* enteric-coated capsules, gut microbiota, intestinal barrier, safety, ulcerative colitis, Wuwei Kushen

## Abstract

Five-flavor *Sophora flavescens* enteric-coated capsules (FSEC), also reported as Wuwei Kushen or composite Sophora colon-soluble capsules, are used in China for ulcerative colitis (UC) associated with intestinal damp-heat, bloody diarrhea, and mucosal injury. This critical narrative Review evaluates the traditional rationale, product identity, material basis, pharmacological evidence, clinical findings, and safety of FSEC. PubMed, Web of Science, ScienceDirect, CNKI, Wanfang, and reference lists were searched through May 2026 and selectively updated through 18 August 2026. The Four Pillars of Best Practice in Ethnopharmacology and the ConPhyMP guidelines informed appraisal of identity, characterization, dose, and evidence attribution. The defined five-material product contains *S. flavescens*, *Sanguisorba officinalis*, Indigo naturalis, *Bletilla striata*, and *Glycyrrhiza uralensis*. Complete-formulation experiments support effects on microbial short-chain-fatty-acid signaling, GPR43, NLRP3/STAT3, AGE-RAGE/NF-κB, epithelial barrier integrity, and ferroptosis-related changes. Constituent studies broaden biological plausibility but cannot establish the efficacy or safety of the complete product. Older randomized trials and meta-analyses suggest short-term benefit, whereas recent single-center cohorts provide provisional adjunctive evidence because of nonrandomized designs and incomplete batch reporting. FSEC is therefore a plausible colon-targeted ethnopharmacological intervention, but confirmatory studies require chemically defined batches, verified release and exposure, internationally accepted UC endpoints, prospective multicenter designs, and active monitoring for hepatic, cardiopulmonary, electrolyte, and interaction risks.

## Introduction

Ulcerative colitis (UC) is a chronic, relapsing inflammatory disease beginning in the rectum and extending proximally through the colon in a continuous pattern. Bloody diarrhea, urgency, abdominal pain, fatigue, and impaired quality of life reflect a disease process that is clinically visible at the mucosal surface but biologically distributed across epithelial, immune, vascular, neural, and microbial compartments. Its worldwide burden has expanded beyond historically high-incidence regions, and health systems in newly industrialized countries increasingly face both incident disease and cumulative long-term care needs (Ng et al., 2017; Gros and Kaplan, 2023; Le Berre et al., 2023). The therapeutic objective has accordingly shifted from episodic symptom relief toward sustained control of symptoms, endoscopic inflammation, steroid exposure, hospitalization, surgery, and disability.

Contemporary treatment includes 5-aminosalicylic acid (5-ASA), corticosteroids, thiopurines, biologics directed at tumor necrosis factor, integrins, interleukin-12/23 or interleukin-23, and small molecules targeting Janus kinases or sphingosine-1-phosphate receptors. These options have improved outcomes, yet primary nonresponse, secondary loss of response, adverse effects, access barriers, and therapeutic sequencing remain important. Network meta-analyses show that several advanced therapies are effective in moderate-to-severe UC, but indirect comparisons do not identify a universally superior drug for an individual patient (Baumgart and Le Berre, 2021; Burr et al., 2022; Shehab et al., 2025). Living guidance therefore emphasizes disease severity, prior exposure, safety, patient preference, and shared decision-making rather than a single fixed ladder (Singh et al., 2024).

The expansion of therapeutic choice has also raised the standard by which complementary and traditional interventions should be judged. A reduction in a nonvalidated symptom score is no longer sufficient when endoscopic response, histologic activity, fecal calprotectin, steroid-free remission, and durability can be assessed. Fecal calprotectin predicts relapse risk, and persistent histologic inflammation may forecast future clinical relapse even in endoscopic remission (Costa et al., 2005; Li J. et al., 2019; George et al., 2023). Treat-to-target strategies and international consensus are moving toward comprehensive disease control that integrates symptoms, biomarkers, endoscopy, quality of life, and disease modification (Ungaro et al., 2019; Schreiber et al., 2024). Any evaluation of an herbal product for UC must therefore connect traditional goals to endpoints that are interpretable within this modern framework.

Five-flavor *Sophora flavescens* enteric-coated capsules (FSEC) are a Chinese patent medicine described in the literature under several English names, including Wuwei Kushen, Fufangkushen colon-coated capsule (FCC), and composite Sophora colon-soluble capsule (CSCC). The intended indication is commonly framed as active UC with an intestinal damp-heat pattern and manifestations such as bloody or mucous stool, tenesmus, abdominal discomfort, and urgency. The dosage form is designed to delay release until the distal gut. These features make FSEC relevant to ethnopharmacology because traditional pattern logic, a multi-material pharmaceutical product, colon-targeted delivery, contemporary mechanistic experiments, and clinical studies converge in a single intervention.

The evidence base is nevertheless easy to overstate. Studies of matrine, oxymatrine, kurarinone, Indigo naturalis, or another Kushen-containing prescription are not experiments on FSEC. Likewise, network pharmacology may generate hypotheses about targets, but cannot demonstrate that a named compound reaches diseased mucosa at an active concentration. Differences in source material, processing, extraction, excipients, coating, release behavior, and dose may alter both benefit and harm. Reviews that pool these evidence units without a clear hierarchy risk turning chemical possibility into product-level certainty (Chen M. et al., 2020; Liu et al., 2022; Zhang et al., 2022).

This Review addresses that problem by asking five linked questions: what exactly is the FSEC product; how does its traditional formulation logic relate to UC symptoms; which constituents and pharmaceutical properties plausibly determine exposure; which mechanisms and outcomes have been demonstrated with the complete formulation; and what clinical and safety conclusions are defensible? The analysis follows the Four Pillars of Best Practice in Ethnopharmacology—traditional context, botanical and material identity, chemical characterization, and biological relevance—and uses ConPhyMP to identify reporting gaps that affect reproducibility (Heinrich et al., 2022). The aim is not to endorse or dismiss FSEC, but to define what is known, what is inferred, and what must be tested next. Figure 1 summarizes the formulation, proposed mechanisms, clinical evidence, and safety considerations.

**FIGURE 1:**
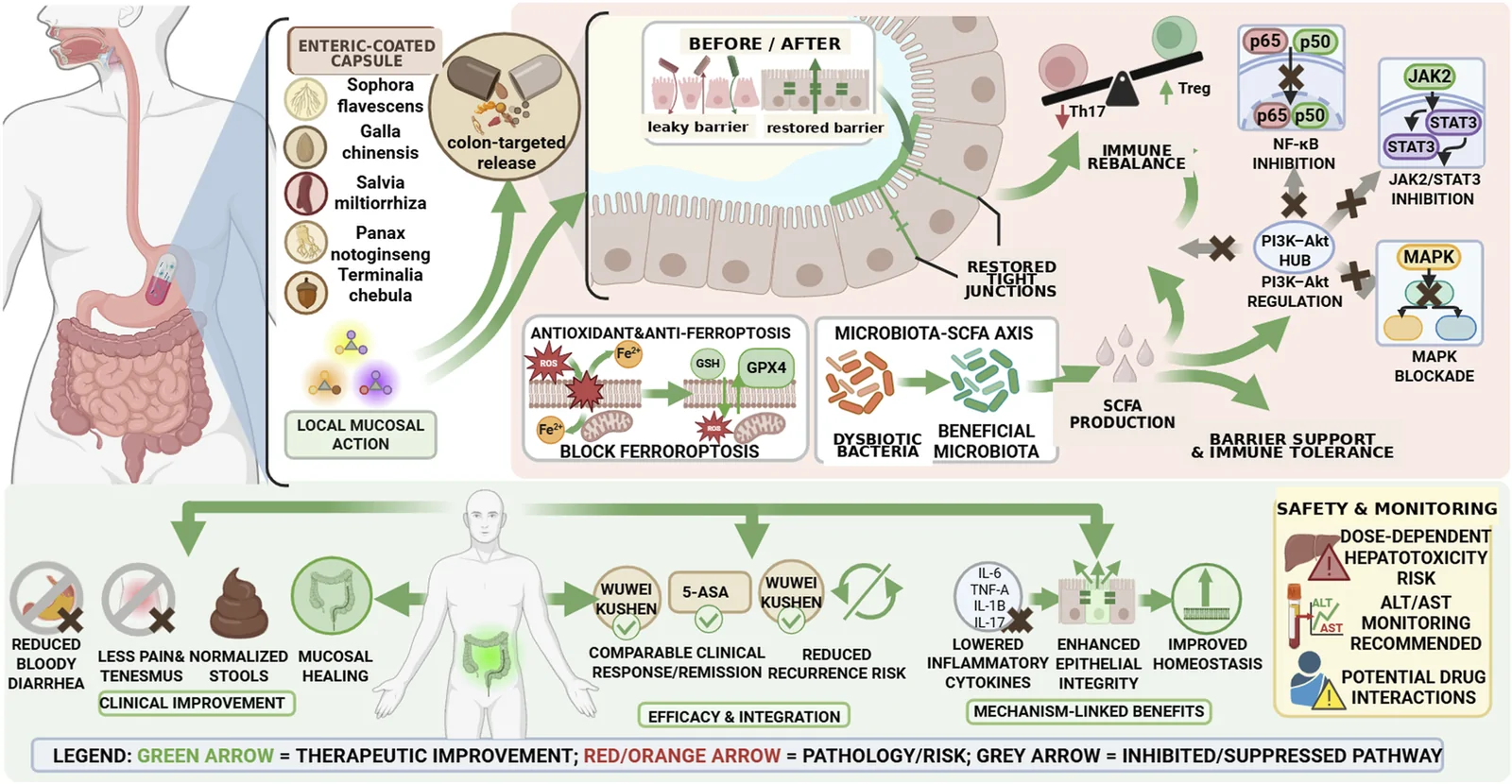
FSEC in ulcerative colitis: product identity, evidence levels, proposed mechanistic modules, clinical evidence, and safety monitoring. Solid elements denote complete-product evidence; dashed elements denote supportive or provisional evidence. FSEC, Five-flavor Sophora flavescens enteric-coated capsules; GPR43, G protein-coupled receptor 43; ILC3, group 3 innate lymphoid cell; NF-κB, nuclear factor kappa B; NLRP3, NOD-like receptor family pyrin domain-containing 3; SCFA, short-chain fatty acid; STAT3, signal transducer and activator of transcription 3.

## Review methods

### Search strategy and update

This work is a critical narrative Review rather than a prospectively registered systematic review. PubMed, Web of Science, ScienceDirect, China National Knowledge Infrastructure, Wanfang Data, and the reference lists of eligible publications were searched through May 2026. A targeted update was performed through 18 August 2026 to identify recent complete-formulation pharmacology, clinical cohorts, safety studies, UC treatment guidance, and product-relevant constituent research. English and Chinese search terms combined ulcerative colitis, inflammatory bowel disease, colitis, Wuwei Kushen, Five-flavor Sophora, Fufangkushen, composite Sophora colon-soluble capsule, *S. flavescens*, Kushen, matrine, oxymatrine, kurarinone, Indigo naturalis, *Sanguisorba officinalis*, *Bletilla striata*, and *Glycyrrhiza uralensis*.

Eligible evidence included randomized or comparative clinical studies of a recognizable FSEC/FCC/CSCC product; systematic reviews and meta-analyses containing those interventions; complete-formulation animal, cell, network-assisted, metabolomic, or microbiome studies; and constituent or component studies with a direct connection to UC biology, pharmacokinetics, quality control, or safety. Broad UC reviews and guidelines were included to establish current clinical endpoints. Reports were excluded from product-level conclusions when the prescription was materially different, the plant identity was unclear, the preparation could not be linked to the named product, or the measured outcome did not inform UC pharmacology, efficacy, or safety.

### Evidence classification and critical synthesis

Evidence was classified by experimental unit before interpretation. Tier 1 comprised the complete five-material enteric-coated formulation or a clearly equivalent named product. Tier 2 comprised a single authenticated component, a defined extract, or a quantified compound that occurs within the formulation. Tier 3 comprised adjacent evidence, such as other Qingre-Chushi prescriptions, general herbal-delivery studies, disease biomarkers, or mechanistic reviews. Tier 1 findings can support product-level hypotheses; Tier 2 findings support plausibility or identify hazards; Tier 3 findings supply context. Neither Tier 2 nor Tier 3 was treated as proof that FSEC produces the same exposure or clinical effect.

Clinical evidence was appraised for randomization, concealment, blinding, comparator, product identity, diagnostic and pattern criteria, sample size, prespecification of outcomes, intention-to-treat analysis, duration, missing data, safety ascertainment, and use of validated clinical, endoscopic, histologic, or biomarker endpoints. Meta-analyses were assessed for formulation heterogeneity, route, comparator, trial overlap, risk of bias, and whether pooled outcomes represented remission or a broadly defined ‘effective rate.’ Preclinical studies were considered in relation to model choice, preventive versus therapeutic dosing, positive and vehicle controls, dose relevance, batch description, chemical characterization, replication, and the distinction between target association and causal validation.

### Ethnopharmacology and ConPhyMP appraisal

The Four Pillars framework was applied as an organizing discipline. Traditional context required a defined indication and an explanation of how each material contributes to the prescription logic. Identity required accepted scientific names, medicinal parts, processing status, and recognition that Indigo naturalis is a processed material rather than a single botanical species. Chemical and pharmaceutical characterization required, where available, chromatographic fingerprints, quantitative markers, contaminants, stability, processing information, and enteric-release performance. Biological relevance required doses and exposure conditions that could reasonably inform use of the marketed product.

ConPhyMP was used as an appraisal framework, not claimed as completed batch certification. This Review did not collect plants, prepare capsules, or test a new batch; it cannot retrospectively supply voucher specimens, extraction yields, certificates of analysis, or release profiles missing from published articles. Instead, the framework identifies information relevant to future FSEC studies: botanical authentication, medicinal parts, processing, extraction or preparation procedures, excipients, marker assays, chromatographic fingerprints, contaminant limits, dose calculation, storage, and dissolution or disintegration across physiologic pH conditions (Heinrich et al., 2022).

### Limits of the review method

The search was broad but not conducted with duplicate independent screening, a registered protocol, formal risk-of-bias instruments, or *de novo* quantitative synthesis. Chinese-language indexing and inconsistent English product names create an additional retrieval risk. The Review therefore does not provide pooled effect estimates and does not grade certainty using GRADE. Its strength is a transparent evidence hierarchy and an updated integration of product identity, mechanisms, clinical data, and safety; its limitation is that selection and interpretation remain narrative. Conclusions are deliberately calibrated to study design, and unresolved preparation or batch information is reported as uncertainty rather than reconstructed by assumption.

## Ethnopharmacological context and product identity

### Traditional indication and therapeutic logic

UC has no exact one-to-one equivalent in classical Chinese medicine, but its manifestations overlap with categories such as dysentery, diarrhea, intestinal wind, and bloody stool. In active disease, the pattern commonly labeled intestinal damp-heat emphasizes frequent urgent stool, mucus or blood, abdominal pain, tenesmus, a sensation of incomplete evacuation, and systemic or tongue and pulse findings interpreted as heat and damp accumulation. This pattern language is not a substitute for endoscopic diagnosis. Rather, it functions as a treatment-selection system layered onto a biomedical diagnosis and is relevant because FSEC trials frequently restrict enrollment or report response using such criteria (Tong et al., 2010; Gong et al., 2012).

Within that logic, Kushen, the dried root of *S. flavescens*, is the principal heat-clearing and damp-drying material. Diyu, the root of *S. officinalis*, is traditionally used to cool the blood and reduce bleeding. Qingdai, Indigo naturalis, contributes heat-clearing and blood-cooling functions and is particularly associated with bloody inflammatory presentations. Baiji, the tuber of *B. striata*, is used for hemostasis and tissue repair. Gancao, the root and rhizome of *G. uralensis*, is used to harmonize multi-herbal prescriptions and may contribute anti-inflammatory and antispasmodic actions. Table 1 links these roles to representative chemical groups without claiming that traditional function is explained by a single molecule.

**TABLE 1:** Identity, traditional role, representative constituents, and quality priorities for FSEC components.

Chinese materia medica	Scientific source and medicinal part	Traditional role in UC-related symptoms	Representative markers or groups	Quality and safety priorities
Kushen	*Sophora flavescens* Aiton (Fabaceae); dried root	Clears heat and dries dampness; principal material for dysentery-like inflammation	Matrine, oxymatrine, sophoridine, oxysophocarpine; prenylated flavonoids	Species authentication; alkaloid/flavonoid fingerprint; hepatic, neurologic, and cardiovascular safety
Diyu	*Sanguisorba officinalis* L. (Rosaceae); dried root	Cools blood, arrests bleeding, and reduces toxin-related inflammation	Hydrolysable tannins, polyphenols, triterpenoid saponins	Identity and marker panel; quantify variability; document processing and chemical consistency
Qingdai	Indigo naturalis; processed material from pharmacopoeial botanical sources	Clears heat and toxin, cools blood, and targets bloody stool	Indigo, indirubin, tryptanthrin, and related indoles	Source/processing traceability; indigoid assay; hepatic and pulmonary monitoring
Baiji	*Bletilla striata* (Thunb.) Rchb.f. (Orchidaceae); dried tuber	Arrests bleeding and supports tissue or mucosal repair	Glucomannan-rich polysaccharides; phenanthrene/bibenzyl-related compounds	Species authentication; polysaccharide attributes; small-molecule fingerprint; contaminants
Gancao	*Glycyrrhiza uralensis* Fisch. ex DC. (Fabaceae); dried root and rhizome	Harmonizes the prescription; contributes anti-inflammatory and antispasmodic actions	Glycyrrhizin, glycyrrhetinic acid, liquiritin, isoliquiritigenin	Glycyrrhizin assay; duplicate exposure; blood pressure and potassium monitoring

The prescription therefore combines four therapeutic intentions: suppress the inflammatory pattern described as damp-heat; reduce bleeding; protect or repair injured mucosa; and temper the tolerability of the combination. This is more specific than describing FSEC as a generic anti-inflammatory formula. It also creates testable modern hypotheses: stool frequency and rectal bleeding should improve together with mucosal inflammation; barrier recovery should be demonstrable rather than inferred from symptom change; and a hemostatic claim should not conceal ongoing disease activity. Traditional and biomedical outcomes should be measured in parallel, not substituted for each other.

### Nomenclature and the risk of product conflation

FSEC, FCC, CSCC, and Wuwei Kushen have been used for what appears to be the same five-material colon-targeted product lineage, but naming is not uniform across translations and time. ‘Compound Kushen’ is especially hazardous because it can refer to other oral products, injections, or prescriptions with different ingredients and indications. A paper should therefore be considered directly relevant only after its composition, dosage form, or citation chain is checked. Search strategies can be inclusive; evidence synthesis must be restrictive. Product conflation is one reason why favorable findings for Kushen-based medicine cannot automatically be assigned to the defined FSEC capsule (Chen M. et al., 2020; Hou et al., 2022).

The composition used throughout this Review is *S. flavescens*, *S. officinalis*, Indigo naturalis, *B. striata*, and *G. uralensis*. Substitution with Galla chinensis, *Salvia miltiorrhiza*, *Panax notoginseng*, or *Terminalia chebula* would describe a different formula. Scientific names, accepted authorship, family, medicinal part, and processing should be reported at first mention or in a product table; pharmacopoeial identity and quality criteria should be cited where applicable (Chinese Pharmacopoeia Commission, 2020).

### The Four Pillars applied to FSEC

The first pillar, traditional context, is reasonably represented in older clinical studies through the damp-heat indication, but the diagnostic reproducibility of pattern classification is rarely quantified. The second pillar, material identity, is only partly satisfied because product names are often reported without adequate material description, batch information, or raw-material authentication. The third pillar, chemical characterization, is the largest recurrent weakness: many studies report dose and duration without quantitative fingerprints or dissolution performance. The fourth pillar, biological relevance, is mixed because complete-product models exist, yet preventive administration, high doses, or surrogate endpoints may not resemble clinical induction therapy.

These deficits matter mechanistically. If one batch contains more quinolizidine alkaloids and another more indigoids, the two preparations may differ in intestinal exposure, efficacy, hepatotoxicity, and interactions. If the coating releases early, systemic exposure may increase while colonic exposure falls. If a study uses a water extract of one component but the commercial product uses a different process, target engagement may not transfer. Good ethnopharmacology therefore begins with a reproducible intervention. Mechanistic sophistication cannot compensate for an undefined product.

## Material basis, pharmaceutical properties, and quality control

### *Sophora flavescens*: alkaloids and prenylated flavonoids

*S. flavescens* contains quinolizidine alkaloids—including matrine, oxymatrine, sophoridine, oxysophocarpine, and N-methylcytisine—and prenylated flavonoids such as kurarinone and kushenol derivatives. The genus has a wide chemical and traditional-use profile, so a list of compounds detected somewhere in *Sophora* should not be mistaken for the quantitative composition of FSEC (Sun et al., 2022). Alkaloids are often prioritized as markers because they are abundant, pharmacologically active, and safety-relevant. Flavonoids may contribute immune, epithelial, smooth-muscle, and microbial effects; direct activation of large-conductance calcium-activated potassium channels by isolated flavonoids illustrates one possible antispasmodic mechanism, but its contribution after oral FSEC remains unknown (Lee et al., 2018).

The alkaloid fraction is not pharmacokinetically static. Oxymatrine can undergo reductive metabolism to matrine, with microsomal CYP3A4 involvement reported *in vitro* (Liu et al., 2015). Integrated *in vivo* metabolism and network analysis of a defined *S. flavescens* ethyl acetate extract further illustrates how detected metabolites can be used to narrow UC-relevant hypotheses (Chen L. et al., 2020). This interconversion complicates attribution because administered oxymatrine, absorbed oxymatrine, generated matrine, and locally retained material may contribute differently. For a colon-targeted product, plasma concentration alone may underestimate mucosal exposure, whereas high systemic concentrations may be more relevant to toxicity. Simultaneous measurement of parent alkaloids and metabolites in capsule, luminal contents, colonic tissue, portal blood, and systemic plasma would clarify whether the enteric design changes the benefit-risk relationship.

### *Sanguisorba officinalis*, Indigo naturalis, *Bletilla striata*, and licorice

*S. officinalis* contributes hydrolysable tannins, polyphenols, and triterpenoid saponins. These groups are consistent with astringent, hemostatic, anti-inflammatory, and barrier-related hypotheses, but formula-specific contribution remains underdefined. A defined ethyl acetate extract attenuated experimental colitis with changes in PI3K-AKT/NF-κB/STAT3 signaling at single-cell resolution, while *Sanguisorba* saponin research links activity to lipid-metabolism regulation (Li et al., 2023; Yu et al., 2023). These studies support biological plausibility for Diyu; they do not show that the same fractions are present or bioavailable at equivalent levels in FSEC.

Indigo naturalis is a processed blue powder commonly produced from pharmacopoeial plant sources and contains indigo, indirubin, tryptanthrin, and related indole compounds. It is one of the most clinically relevant components because oral Indigo naturalis has shown efficacy in UC and can activate aryl hydrocarbon receptor (AhR)-linked mucosal pathways. A multicenter randomized trial demonstrated short-term efficacy of Indigo naturalis in active UC, but safety concerns have constrained unrestricted use (Naganuma et al., 2018). FSEC contains Indigo naturalis within a lower-dimensional multi-material matrix; this makes AhR activity plausible, but the delivered indigoid dose and comparative risk cannot be assumed without quantitative data.

*B. striata* supplies glucomannan-rich polysaccharides as well as phenanthrene- and bibenzyl-related small molecules. Its traditional tissue-repair role is compatible with mucoadhesion, barrier support, and local delivery. A *Bletilla*-polysaccharide/chitosan thermosensitive mucoadhesive hydrogel has been studied as a rectal nanoparticle carrier in colitis, illustrating the material’s pharmaceutical potential rather than the mechanism of oral FSEC (Zhao et al., 2024). A more recent glucomannan study reported barrier, inflammatory, and microbiota effects in experimental colitis (Sun et al., 2025). Both are supportive component-level evidence; neither establishes the dose or function of *Bletilla* polysaccharide released from the marketed capsule.

*G. uralensis* contains glycyrrhizin, glycyrrhetinic acid, liquiritin, isoliquiritigenin, and numerous other constituents. Anti-inflammatory and smooth-muscle effects make licorice a plausible harmonizing component, while microbial conversion of glycyrrhizin to active metabolites makes gut ecology relevant to exposure. The same pathway creates predictable risk: mineralocorticoid-like effects can cause sodium retention, edema, hypertension, hypokalemia, and arrhythmia. The material must therefore be represented in both mechanism and safety models; describing it only as a benign excipient would be pharmacologically inaccurate (Yoshino et al., 2021).

### Microbial transformation and colonic bioavailability

Many herbal glycosides and polyphenols reach the colon incompletely absorbed and are transformed by microbial enzymes. Human gut bacteria can hydrolyze flavonoid glycosides, and the aglycones can differ from their glycosides in permeability, metabolism, and biological activity (Bokkenheuser et al., 1987; Amaretti et al., 2015; Xie et al., 2022; Zhang et al., 2020). FSEC should therefore be viewed as a time-dependent chemical system: manufacturing defines the administered mixture, enteric release defines where it becomes available, microbial and host metabolism generate secondary mixtures, and inflamed mucosa determines local uptake. Interindividual variation in microbiota, transit, pH, disease extent, and concomitant medication may alter this sequence.

The enteric or colon-soluble coating is intended to protect the contents during upper gastrointestinal transit and increase delivery to the distal bowel. That intention is not proof of site-selective release. Patients with active UC may have accelerated transit and altered luminal pH; fed state, proton-pump inhibitors, capsule storage, coating thickness, and batch variability may also affect disintegration. Product research should therefore report compendial and biorelevant dissolution across sequential gastric, small-intestinal, and colonic conditions, together with intact-capsule stability and, ideally, imaging or marker-based confirmation of release in humans. Figure 2 distinguishes the delivery hypothesis from demonstrated exposure.

**FIGURE 2:**
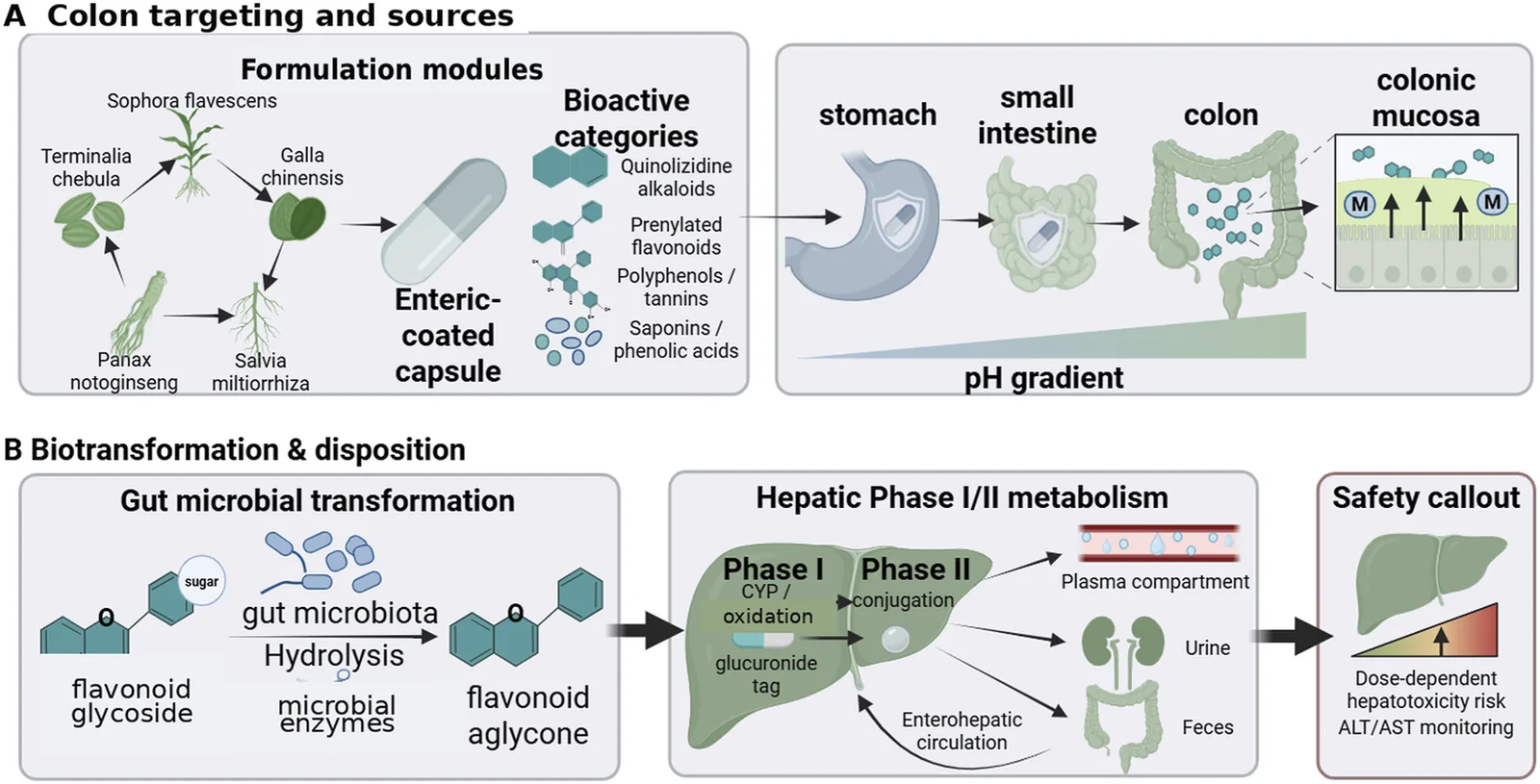
Proposed colonic delivery and disposition of FSEC. **(A)** Formulation sources, bioactive categories, colonic release, and mucosal exposure. **(B)** Microbial transformation, hepatic phase I/II metabolism, systemic distribution, enterohepatic circulation, and elimination, with potential hepatotoxicity and ALT/AST monitoring.

A complete pharmacokinetic program would combine targeted quantification of marker compounds with untargeted metabolomics. Measurements should include alkaloids, indigoids, licorice metabolites, selected tannin or saponin products, and microbiota-derived metabolites such as short-chain fatty acids (SCFAs). Sampling of plasma alone cannot resolve whether efficacy is driven by local luminal action, mucosal tissue exposure, or systemic immunomodulation. Conversely, fecal abundance does not prove epithelial target engagement. Time-resolved analysis across capsule, lumen, mucosa, portal circulation, and systemic circulation is needed to connect dose, release, metabolism, mechanism, and toxicity.

### Quality control as a prerequisite for causal inference

A pragmatic FSEC specification should combine identity, assay, fingerprint, performance, and safety tests. Identity should cover each botanical or processed material and medicinal part. Assay should quantify a panel rather than one nominal marker: representative quinolizidine alkaloids, prenylated flavonoids, indigoids, *Sanguisorba* tannin or saponin markers, *Bletilla* polysaccharide attributes, and glycyrrhizin-related compounds. Chromatographic or spectrometric fingerprint similarity can capture broader consistency. Performance testing should include disintegration and dissolution. Safety testing should include pesticides, heavy metals, mycotoxins, microbial limits, adulterants, and residual solvents where relevant.

Batch characterization is not a ceremonial supplement to biology. It determines whether another laboratory can reproduce the intervention, whether two clinical trials tested comparable products, and whether an adverse reaction can be traced. Marker ranges should be justified by manufacturing capability and pharmacological relevance, not selected only because standards are commercially convenient. Ideally, a reference batch would link quantitative composition to mucosal exposure and a multidimensional biological signature. Until such data exist, cross-study differences should be interpreted as possible product heterogeneity rather than automatically attributed to population or experimental noise.

## Pharmacological evidence relevant to ulcerative colitis

### Complete-formulation evidence and inflammatory signaling

The most informative mechanistic studies administer the recognizable complete formulation. Integrative pharmacology and microbial-network work associated CSCC with inflammatory and microbial pathways and then tested selected predictions experimentally (Ding et al., 2020). In dextran sulfate sodium (DSS) colitis, complete-product studies have reported improved disease activity, colon injury, inflammatory mediators, barrier proteins, microbiota-related metabolites, or immune-cell endpoints. These experiments move beyond ingredient speculation because the biological system encounters the combined preparation. Their main limitations are incomplete batch characterization, uncertain equivalence across product names, small animal samples, and frequent use of acute chemically induced models.

Recent complete-formulation reports provide a more coherent mechanistic network. CSCC increased gut microbiota-derived butyrate and was linked to GPR43 signaling and NCR-positive group 3 innate lymphoid cells (ILC3) in murine colitis (Chen et al., 2023). Wuwei Kushen Changrong capsule inhibited NLRP3 inflammasome and STAT3-related signaling (Chen et al., 2024). An integrative study implicated AGE-RAGE/NF-κB, while another experimental protocol focused on ferroptosis-related changes (Hu et al., 2026; Wang et al., 2026). Together, these findings support multi-compartment activity, but convergence among papers is not equivalent to a validated causal chain.

Three questions determine how much confidence these studies deserve. First, did the experiment test an authenticated batch and report a clinically interpretable dose? Second, were pathway results confirmed by perturbation—such as receptor antagonism, genetic manipulation, metabolite rescue, or microbiota transfer—rather than correlation alone? Third, did the dosing schedule model treatment after colitis was established rather than prevention before injury? Without these controls, a lower cytokine concentration may simply follow reduced tissue damage, and a shifted microbiome may be consequence rather than mediator. The evidence supports hypotheses suitable for translational testing, not a claim that every listed pathway is an independent therapeutic target of FSEC.

NF-κB integrates signals from microbial products, cytokine receptors, oxidative stress, and damaged cells, promoting transcription of inflammatory mediators. NLRP3 inflammasome activation can amplify interleukin-1β and epithelial injury, whereas STAT3 has context-dependent roles in immune activation and epithelial repair. The AGE-RAGE axis can intensify oxidative and NF-κB signaling. Complete-product results involving NLRP3/STAT3 and AGE-RAGE/NF-κB are therefore biologically coherent (Chen et al., 2024; Hu et al., 2026). However, pathway protein changes in bulk colon tissue cannot identify the responsible cell type or distinguish primary target engagement from normalization secondary to reduced disease severity.

Constituent research supplies orthogonal support. Matrine has been linked to TLR4/NF-κB/MAPK signaling in inflammatory cell and intestinal-injury models, while experiments in DSS or TNBS colitis report effects on cytokines, oxidative stress, barrier integrity, and microbial structure (Li P. et al., 2019; Mao et al., 2024a; Mao et al., 2024b; Yao H. et al., 2021). Reviews of herbal bioactives place such findings within a wider range of delivery and chronic intestinal-inflammation strategies (Harwansh et al., 2024). Network-pharmacology analyses of *S. flavescens* predict inflammatory targets, but prediction confidence depends on compound databases, oral-bioavailability filters, and target annotation (Zhu and Hou, 2021). These data justify targeted assays in FSEC-treated systems; they cannot establish matrine as the sole or dominant mediator of the full product.

PI3K/Akt and JAK/STAT signaling connect growth, survival, metabolic stress, cytokine responses, and T-cell differentiation. Reviews of natural products and traditional Chinese medicine identify these pathways as recurrent UC nodes (Niu and Zhang, 2025; Long et al., 2025). Kurarinone and total *S. flavescens* flavonoids have been associated with Th17/Treg homeostasis, and defined *Sanguisorba* preparations have affected PI3K-AKT/NF-κB/STAT3-related programs (Li et al., 2023; Li et al., 2022). Because the same pathways operate in epithelial repair and host defense, the desired outcome is restoration of context-appropriate signaling, not indiscriminate inhibition.

### Mucosal immunity and epithelial barrier

The colonic immune system must tolerate food and commensals while responding to invasive organisms. UC disrupts this balance through altered antigen sensing, epithelial alarmins, myeloid activation, effector T-cell expansion, regulatory-cell dysfunction, and changes in innate lymphoid cells. The reported butyrate-GPR43-NCR-positive ILC3 axis is attractive because it connects microbial metabolism to mucosal immunity and epithelial support (Chen et al., 2023). Stronger causal evidence would require depletion or blockade experiments showing that loss of GPR43 signaling, butyrate production, or the relevant ILC3 population attenuates FSEC benefit.

Th17 cells contribute to antimicrobial defense but can intensify chronic inflammation, whereas regulatory T cells restrain excessive responses. Total *S. flavescens* flavonoids and kurarinone improved Th17/Treg-related endpoints in experimental colitis, and kurarinone has also been linked to broader regulation of T-cell immunity (Li et al., 2022; Pan et al., 2025). These are constituent-level findings. In FSEC, indigoids may simultaneously activate AhR, microbial metabolites may influence G-protein-coupled receptors, and licorice or tannin fractions may alter innate responses. Cell-resolved studies are needed to determine whether the combined product produces additive, antagonistic, or simply overlapping effects.

Indigo naturalis offers the clearest clinical bridge for AhR-related reasoning. Indole compounds can act as AhR ligands, supporting epithelial defense and interleukin-22-linked repair. Clinical efficacy of the single processed material makes this pathway more than a database prediction (Naganuma et al., 2018; Wu et al., 2025). Yet FSEC-specific questions remain: the content and release of indigo and indirubin, the balance between local activation and systemic toxicity, and interactions with other formula components. AhR reporter assays and targeted indigoid pharmacokinetics should be performed on the same characterized FSEC batch used in efficacy experiments.

The epithelial barrier is a dynamic system of mucus, antimicrobial peptides, tight junctions, cell renewal, and immune-microbial communication. In UC, goblet-cell dysfunction and alterations in MUC2 permit closer bacterial contact, while epithelial death and disrupted tight junctions increase permeability. MUC2 and bacterial factors related to mucus utilization are therefore plausible therapeutic targets, but their normalization requires spatial and functional evidence rather than a single bulk-protein measurement (Yao D. et al., 2021). Complete-product studies reporting recovery of ZO-1, occludin, or histologic architecture support barrier protection, although permeability assays and durability after treatment withdrawal are less consistently available.

Constituent-level data reinforce this theme. Matrine improved gut barrier-related endpoints in experimental colitis; kushenol I was associated with barrier preservation and microbiota optimization; and *Bletilla* glucomannan affected barrier, inflammatory, and microbial outcomes (Sun et al., 2025; Yao H. et al., 2021; He et al., 2025). These materials may act through distinct physicochemical and cellular routes, including mucoadhesion, metabolite production, and epithelial signaling. A factorial or component-deletion design using chemically matched preparations would be more informative than simply testing five isolated compounds at arbitrary concentrations.

Secretory immunoglobulin A (sIgA) forms an adaptive barrier that coats microbes and shapes community structure without provoking destructive inflammation. It is connected to epithelial transport, B-cell differentiation, and microbial ecology (Mantis et al., 2011; Pietrzak et al., 2020). Some herbal-colitis studies report changes in mucosal immunoglobulins, but sIgA should not be interpreted in isolation: increased concentration could reflect restored secretion, altered luminal water, or an ongoing immune response. Future FSEC studies should combine sIgA quantity, bacterial coating patterns, mucus structure, permeability, and spatial microbiota analysis.

### Oxidative stress, ferroptosis, and microbiota

Reactive oxygen species contribute to lipid peroxidation, mitochondrial dysfunction, and epithelial injury in active UC. Ferroptosis, an iron-dependent form of regulated cell death associated with phospholipid peroxidation and impaired antioxidant defense, has therefore emerged as a mechanistic target for traditional medicine (Guo et al., 2024). Oxymatrine improved inflammatory and ferroptosis-related measures in experimental colitis, and complete FSEC has been studied using similar endpoints (Wang et al., 2026; Gao et al., 2024). A persuasive demonstration would include orthogonal markers, iron and lipid-peroxide measures, morphological or functional confirmation, and rescue by a specific ferroptosis inhibitor.

The same biology creates a safety paradox. Matrine has produced hepatocyte oxidative injury, mitochondrial apoptosis, suppression of Nrf2-mediated defense, disruption of Nrf2/GPX4 signaling, and ferroptosis-related toxicity in experimental systems (You et al., 2019; Wang et al., 2023). Transcriptomic and metabolomic analyses also identify pathways associated with matrine hepatotoxicity, and more recent work implicates abnormal cholesterol-synthesis signaling through DHCR7 (Rao et al., 2022; Tao et al., 2025). Consequently, an antioxidant or anti-ferroptotic effect in inflamed colon cannot be generalized to liver, dose, or duration. Tissue exposure and therapeutic window are central.

UC-associated dysbiosis is heterogeneous but commonly includes reduced functional resilience, disturbed metabolite production, and altered interactions with mucus and immunity. Herbal mixtures can change substrate availability, antimicrobial pressure, transit, inflammation, and host secretion, all of which reshape microbial communities. Matrine and *S. flavescens* flavonoids altered microbial and metabolite networks in colitis models, while broader reviews position intestinal microecology as a bridge among flora, barrier function, and immunity (Li P. et al., 2019; Shao et al., 2021; Wang et al., 2024).

The complete-product butyrate-GPR43 finding is therefore important because it links a microbial function to a host receptor rather than relying only on taxonomic abundance (Chen et al., 2023). Even so, higher fecal butyrate can reflect increased production, reduced absorption, or altered transit. Demonstrating mediation would require absolute metabolite quantification, isotope tracing or functional assays, and interventions such as antibiotics, gnotobiotic transfer, receptor blockade, or defined microbial consortia. Baseline diet, cage effects, sequencing pipeline, and compositional statistics should be prespecified. Human translation should focus on reproducible functions rather than a universal list of ‘beneficial bacteria'.

### Synergy and evidence interpretation

Multi-component prescriptions are often described as synergistic, but synergy has a quantitative meaning: the combined effect exceeds an explicit additivity model at defined doses. FSEC could plausibly combine direct anti-inflammatory activity, AhR signaling, microbial transformation, mucoadhesion, hemostatic effects, and tolerability modification. It could also contain antagonistic constituents, alter absorption, or concentrate shared toxicities. Network diagrams do not distinguish these possibilities. Claims of synergy should therefore be based on response surfaces, isobolographic or model-based interaction analysis, component-deletion preparations, and chemical confirmation that each test condition is comparable.

A useful sequence would test the complete batch, each component, rational subcombinations, and a reconstituted mixture at exposure-matched concentrations. Mechanistic perturbations could then identify which component is necessary or sufficient for a given endpoint. Such work should avoid reducing the product to one favored marker: a marker may be analytically convenient without mediating efficacy. Systems approaches are valuable when they generate falsifiable hypotheses and integrate pharmacokinetics; they are weak when numerous predicted targets are treated as confirmation. Table 2 summarizes the present evidence by experimental unit and the limits of inference.

**TABLE 2:** Evidence hierarchy for proposed FSEC mechanisms.

Evidence unit	Representative sources	UC-relevant findings	Interpretive status
Complete FSEC/CSCC formulation	Ding et al. (2020); Chen et al. (2024); Hu et al. (2026); Wang et al. (2026); Chen et al. (2023)	Microbial butyrate/GPR43/NCR + ILC3; NLRP3/STAT3; AGE-RAGE/NF-κB; barrier and ferroptosis-related changes	Direct product-level experimental evidence; mostly preclinical, acute-model, and incompletely batch-characterized
Matrine and oxymatrine	Li et al. (2019b); Mao et al. (2024a); Mao et al. (2024b); Yao H. et al. (2021); Gao et al. (2024)	Inflammatory signaling, oxidative stress, barrier, microbiota, and ferroptosis-related pathways	Strong constituent-level support; not equivalent to full-product efficacy or safety
Kurarinone and related flavonoids	Li et al. (2022); Pan et al. (2025); He et al. (2025)	Th17/Treg and T cell regulation; barrier preservation; microbiota	Supportive *in vivo* evidence; exposure within FSEC is insufficiently quantified
Indigoids	Naganuma et al. (2018); Wu et al. (2025)	AhR-linked mucosal immune plausibility and clinical activity of Indigo naturalis	Clinically relevant component evidence; benefit must be balanced against hepatic and pulmonary risk
*Sanguisorba* tannins/saponins	Li et al. (2023); Yu et al. (2023)	PI3K-AKT/NF-κB/STAT3 and lipid-metabolism-related findings	Supportive component evidence; formula-specific contribution remains uncertain
*Bletilla* polysaccharides	Zhao et al. (2024); Sun et al. (2025)	Mucoadhesion/delivery; barrier, inflammation, and microbiota	Supportive component and material-science evidence; oral FSEC exposure underdefined
Licorice constituents	Yoshino et al. (2021)	Anti-inflammatory plausibility and microbial metabolism	Supportive; pseudoaldosteronism risk requires dose and duration control

## Clinical evidence

### Randomized and comparative studies of the complete product

The most frequently cited direct clinical evidence includes older multicenter trials of FCC or CSCC in active UC. A multicenter randomized single-blind controlled study reported efficacy for CSCC, and a later double-blind, double-dummy randomized study compared FCC with mesalazine in 320 participants (Tong et al., 2010; Gong et al., 2012). The latter design is notable because double-dummy masking can preserve blinding when dosage forms differ. Short-term clinical and mucosal outcomes were broadly comparable between groups over approximately 8 weeks. These trials establish more credible product-level evidence than uncontrolled case series, but their age, reporting standards, limited follow-up, and incomplete batch characterization constrain current interpretation.

The comparator question is clinically important. Demonstrating no statistically significant difference from mesalazine does not by itself prove equivalence unless an equivalence or noninferiority margin, adequate power, adherence, analysis population, and confidence interval were prespecified. A short induction comparison also cannot establish maintenance efficacy or long-term safety. Furthermore, traditional-pattern restrictions may enrich a responsive population but reduce generalizability. Future trials should retain pattern information as a stratification or effect-modification variable while ensuring that all patients meet contemporary clinical and endoscopic diagnostic criteria.

### Evidence synthesis and recent clinical studies

A systematic review of Kushen-based traditional Chinese medicine found signals of efficacy and acceptable short-term safety, while a review focused on Five-Flavor *S. flavescens* enteric-coated capsules pooled randomized trials and reported favorable results on clinical effective rate and traditional-pattern outcomes (Chen M. et al., 2020; Hou et al., 2022). Chinese patent medicine and Qingre-Chushi network meta-analyses have also ranked some Kushen-containing interventions favorably (Sun et al., 2021; Zhang et al., 2021). These syntheses increase statistical precision but cannot repair bias in the included trials or convert different formulations into a single intervention.

Several sources of heterogeneity are clinically consequential: monotherapy versus add-on use; oral versus enema delivery; active comparator versus usual care; mild-to-moderate versus broader severity; damp-heat-selected versus unselected patients; variable treatment duration; and outcomes ranging from Mayo score or endoscopy to composite ‘effective rate.’ Small-study effects and selective publication may exaggerate benefit. Network rankings can be particularly unstable when evidence loops are sparse and products are connected only indirectly. The appropriate conclusion is a signal requiring modern confirmation, not a definitive hierarchy over 5-ASA or advanced therapy.

Recent work has expanded the evidence base but not eliminated design limitations. A single-center study evaluated FSEC in combination with vedolizumab during induction and reported favorable week-14 clinical and endoscopic signals; longer-term findings were sensitive to analysis population, with a per-protocol signal not confirmed in intention-to-treat analysis (Li et al., 2026). Nonrandom treatment allocation can reflect baseline severity, clinician preference, affordability, prior response, and adherence. Even sophisticated adjustment cannot remove unmeasured confounding, and missing data can differentially affect apparent durability.

Observational studies are still valuable for feasibility, adherence, dosing, subgroup hypotheses, rare harms, and outcome selection. They can characterize how FSEC is used with mesalazine or biologics and identify whether traditional-pattern status predicts response independently of baseline inflammation. However, an adjunctive cohort cannot prove pharmacological additivity. A prospective randomized factorial or add-on trial is needed to separate the effect of FSEC from concurrent therapy, regression to the mean, and differential follow-up. Table 3 presents clinical evidence according to design, product specificity, and the strongest defensible inference.

**TABLE 3:** Critical summary of clinical evidence.

Evidence type	Main finding	Strength	Principal limitations	Defensible interpretation
Older multicenter randomized trials	Short-term clinical and mucosal outcomes improved; a 320-participant double-dummy trial reported results broadly comparable with mesalazine	Direct randomized product-level evidence	Short follow-up; older reporting standards; incomplete batch data; not designed to establish long-term equivalence	Supports short-term plausibility, not superiority, durable benefit, or replacement of standard care
FSEC-focused meta-analysis	Favorable pooled clinical effective rate and traditional-pattern outcomes	Aggregates product-focused trials	Small trials; nonstandard outcomes; incomplete bias reporting; product/reporting heterogeneity	Preliminary support requiring a modern prospectively registered trial
Broader Kushen/Chinese patent medicine meta-analyses	Some Kushen-containing interventions ranked favorably	Broad comparative context	Different prescriptions, routes, comparators, and indirect network links	Exploratory context; not a product-specific efficacy estimate
2026 vedolizumab plus FSEC cohort	Favorable week-14 response signals; longer-term result sensitive to analysis population	Recognizable clinical and endoscopic endpoints	Single center; nonrandomized; confounding; missing-data sensitivity; limited safety power	Provisional adjunctive evidence; not proof of additive efficacy
Indigo naturalis RCT	Short-term efficacy in active UC	Randomized component-level clinical evidence	Different intervention, dose, and safety context	Supports indigoid/AhR plausibility but cannot be assigned to FSEC

### Ingredient-level evidence and clinical endpoints

Indigo naturalis has stronger direct UC evidence than most individual FSEC components. In a multicenter randomized controlled trial, it improved clinical outcomes in active UC, supporting the therapeutic relevance of indigoid/AhR biology (Naganuma et al., 2018). Nevertheless, the single material and the five-material capsule differ in dose, processing, release, and safety context. Discontinuation follow-up suggests that some patients may remain in remission after stopping Indigo naturalis, but such observations do not define maintenance strategy for FSEC (Shimada et al., 2024). Ingredient trials should be treated as triangulation, not as hidden product trials.

A confirmatory FSEC program should use outcomes accepted in modern UC research: clinical remission based on rectal bleeding and stool frequency, endoscopic improvement or remission using centrally read validated scores, histologic improvement or remission, fecal calprotectin, C-reactive protein, corticosteroid-free remission, quality of life, hospitalization, colectomy, and sustained response. The primary endpoint and assessment time should match induction or maintenance intent. Traditional syndrome scores can be retained as prespecified secondary outcomes, with assessor training and reliability measurement, allowing the traditional indication to be evaluated rather than erased.

Biomarkers should complement rather than replace patient benefit. Fecal calprotectin can support monitoring and relapse prediction, but thresholds vary with context and assay (Costa et al., 2005; Li J. et al., 2019). Histologic inflammation may identify residual risk when endoscopy appears healed (George et al., 2023). A product that reduces bleeding through astringent or hemostatic action without controlling mucosal inflammation could create apparent symptomatic improvement while disease persists. Trials should therefore require concordance across symptoms, endoscopy, biomarkers, and safety and should report each component rather than only a composite response.

### Position relative to standard care

Current evidence does not justify replacing guideline-directed advanced therapy for moderate-to-severe, steroid-dependent, hospitalized, or high-risk UC with FSEC. The most defensible research positions are a monitored alternative or adjunct in selected mild-to-moderate disease, or an adjunct studied alongside stable conventional therapy. Rescue treatment and stopping rules must be explicit. Comparators should reflect the population: optimized mesalazine for mild-to-moderate disease, placebo on background therapy for an add-on question, or an active noninferiority design only when a clinically justified margin and rescue plan are acceptable.

## Safety evaluation and rational use

### Clinical safety evidence

Older trials and meta-analyses generally describe short-term tolerability as acceptable, but absence of a statistical difference in adverse events is not evidence of absence of uncommon or delayed harm. Many studies were designed for efficacy, followed participants for weeks, used inconsistent causality assessment, and did not prespecify laboratory monitoring. Multi-herbal products can produce gastrointestinal intolerance, hypersensitivity, hepatic injury, electrolyte disorders, blood-pressure changes, cardiopulmonary events, and drug interactions. Safety must therefore be assessed at the product, ingredient, metabolite, and patient levels.

### *Sophora* and Indigo naturalis-related risks

Matrine-related experimental hepatotoxicity includes oxidative stress, mitochondrial apoptosis, Nrf2/GPX4 disruption, ferroptosis, and altered cholesterol synthesis (You et al., 2019; Wang et al., 2023; Rao et al., 2022; Tao et al., 2025). These studies often use exposures that may not match FSEC, so they cannot quantify clinical incidence. They do identify plausible mechanisms, organs, and biomarkers for surveillance. Acute *Sophora* alkaloid poisoning has also produced neurologic and cardiovascular manifestations, emphasizing the consequences of excessive or unregulated exposure (Chow et al., 2025). Standardized alkaloid content and avoidance of duplicate Kushen products are basic safeguards.

Baseline alanine aminotransferase, aspartate aminotransferase, alkaline phosphatase, bilirubin, medication history, alcohol exposure, and liver-disease risk should be documented in clinical trials. Repeat testing should be scheduled early enough to detect emerging injury and triggered by fatigue, anorexia, nausea, dark urine, jaundice, pruritus, or right-upper-quadrant symptoms. Stopping criteria and a structured causality assessment should be prespecified. Pharmacokinetic sampling in suspected cases would help distinguish idiosyncratic injury from excessive exposure or product variability.

Indigo naturalis has been associated with liver injury and pulmonary arterial hypertension, among other adverse events, despite clinical efficacy in UC. The risk may depend on dose, duration, host susceptibility, and product quality (Wu et al., 2025). Ischemic colitis has also been reported during use, complicating evaluation because abdominal pain and bloody stool can resemble a UC flare (Cho et al., 2020). FSEC-specific incidence is unknown and cannot be inferred directly from single-ingredient exposure, but the presence of Indigo naturalis justifies active questioning about dyspnea, chest discomfort, syncope, exercise intolerance, worsening abdominal pain, and changes in bleeding pattern.

### Licorice, interactions, and special populations

Glycyrrhizin metabolites can inhibit 11β-hydroxysteroid dehydrogenase type 2, allowing cortisol to activate mineralocorticoid receptors. Susceptible patients may develop hypertension, edema, hypokalemia, metabolic alkalosis, weakness, and arrhythmia. Risk rises with greater exposure, longer duration, older age, constipation or slowed transit, and concurrent licorice-containing products, although individual susceptibility varies (Yoshino et al., 2021). Baseline and follow-up blood pressure and serum potassium are appropriate in longer courses or higher-risk patients. Diuretics, corticosteroids, digoxin, antiarrhythmics, and other potassium-altering drugs warrant special review.

Potential interactions include pharmacokinetic effects on drug-metabolizing enzymes or transporters, pharmacodynamic addition to hepatotoxic, antihypertensive, diuretic, anticoagulant, sedative, or immunomodulatory agents, and altered absorption caused by tannins or gastrointestinal transit. Direct clinical interaction data for FSEC are sparse. A cautious medication reconciliation should include prescription drugs, over-the-counter medicines, supplements, decoctions, and patent medicines. Duplicate exposure to Kushen, Qingdai, or licorice may be missed if only brand names are recorded.

Pregnancy, breastfeeding, childhood, advanced age, substantial hepatic or renal impairment, serious cardiovascular disease, and active infection require separate evidence and should not be assumed safe from general adult trials. Patients with severe acute UC need urgent guideline-based assessment because delayed escalation can be dangerous. Quality defects, adulteration, contamination, or altered enteric release may convert a familiar formula into a different exposure. Pharmacovigilance should record exact product name, batch, dose, duration, co-medication, onset, dechallenge, rechallenge where ethical, laboratory course, and outcome.

### Monitoring framework

Before treatment, clinicians or investigators should confirm UC activity and severity, the intended role of FSEC, the exact product, and all concomitant therapies. Baseline assessment should include symptoms, blood pressure, complete blood count, liver chemistry, renal function, and electrolytes; pregnancy testing and electrocardiography may be appropriate by context. During treatment, symptom and adherence review should be paired with objective inflammation monitoring rather than waiting for a crisis. New dyspnea, edema, syncope, jaundice, marked weakness, severe abdominal pain, neurologic symptoms, or worsening bleeding should prompt immediate reassessment and possible discontinuation. Table 4 proposes a trial-oriented monitoring schedule, not a substitute for individualized medical care.

**TABLE 4:** Proposed minimum design and safety framework for a confirmatory FSEC trial.

Domain/time	Minimum assessment	Purpose	Prespecified action
Product identity and release	Botanical/material identity, batch, excipients, multi-marker assay, fingerprint, contaminants, stability, sequential-pH dissolution	Reproducibility and exposure definition	Use only material meeting prespecified quality and release criteria; retain samples for investigation
Baseline	UC diagnosis/severity; symptoms; endoscopy; histology; fecal calprotectin; CBC; liver/renal chemistry; electrolytes; blood pressure; medications and herbal products	Eligibility, stratification, safety, and confounding control	Exclude severe acute disease or contraindications; optimize rescue plan
Early treatment	Adherence; stool frequency/bleeding; adverse symptoms; liver chemistry and potassium in risk-based schedule; pharmacokinetic subset	Detect toxicity, confirm exposure, and avoid delayed escalation	Stop/reassess for liver injury, hypokalemia, severe pain, dyspnea, syncope, neurologic or cardiovascular symptoms
Primary endpoint	Clinical remission plus centrally read endoscopic improvement at week 8 or 12; intention-to-treat analysis	Internationally interpretable induction efficacy	Use prespecified missing-data handling and rescue-treatment failure rules
Key secondary endpoints	Histology, fecal calprotectin, CRP, quality of life, traditional-pattern score, steroid-free response, exposure and mechanistic biomarkers	Depth, consistency, and mechanism of response	Control multiplicity; distinguish exploratory from confirmatory endpoints
Maintenance/follow-up	Sustained remission, relapse, hospitalization, colectomy, cumulative adverse events, blood pressure, electrolytes, liver and cardiopulmonary surveillance	Durability and delayed risk	Independent safety review and lot-linked pharmacovigilance

## Discussion

### Principal interpretation and precision medicine

The available evidence positions FSEC between a traditional prescription and a modern colon-targeted multi-component drug. Its formulation logic maps coherently onto bloody inflammatory diarrhea, mucosal injury, and the need for tolerability. Complete-product experiments provide more than generic anti-inflammatory speculation: microbial butyrate/GPR43/ILC3, NLRP3/STAT3, AGE-RAGE/NF-κB, barrier, and ferroptosis-related signals have been reported. Older randomized trials and focused meta-analysis offer preliminary clinical support. However, incomplete chemical characterization, product-name ambiguity, short follow-up, heterogeneous endpoints, and limited independent replication prevent high-certainty conclusions.

The central finding is therefore not that FSEC acts through an exhaustive pathway map. It is that several biologically connected modules—delivery, microbial metabolism, mucosal immunity, epithelial integrity, inflammatory amplification, and redox cell death—are plausible and partly supported at the product level. The evidentiary boundary is equally important: constituent studies of matrine, kurarinone, Indigo naturalis, *Sanguisorba* extracts, *Bletilla* polysaccharides, or glycyrrhizin explain possibilities and hazards, but do not estimate the efficacy of the capsule. This separation should be maintained in abstracts, figures, tables, and conclusions.

UC is molecularly and clinically heterogeneous. Molecular subtyping may distinguish immune programs and predict differential drug response, while precision-medicine reviews emphasize the distance between promising biomarkers and usable treatment-selection tools (Mo et al., 2023; Schreiber et al., 2025; Wetwittayakhlang et al., 2024). FSEC research offers an unusual opportunity to test whether a traditional pattern identifies a reproducible biomedical subgroup. This should be approached prospectively: pattern assessment before treatment, blinded outcome evaluation, biomarkers measured without redefining the subgroup after results are known, and interaction testing between pattern status and randomized treatment.

Candidate effect modifiers include disease extent, baseline fecal calprotectin, histologic activity, prior 5-ASA or biologic exposure, microbial metabolic capacity, mucosal AhR activity, butyrate production, epithelial-barrier signatures, and measured exposure to alkaloids or indigoids. A large untargeted omics panel in a small uncontrolled cohort will overfit. A staged program should first confirm robust pharmacodynamic markers, then validate a limited predictive signature in an independent randomized population. The goal is not to retrofit every responder into a traditional or molecular category, but to identify a subgroup that changes the treatment effect and can be recognized before therapy.

### Translational roadmap and safety development

The first development priority is a reference product dossier. It should document botanical and material identity, batch, source and processing, excipients, quantitative multi-marker assays, chromatographic fingerprints, contaminants, stability, and enteric-release performance. The second is a linked exposure study measuring capsule content, dissolution, luminal and mucosal markers, microbial metabolites, and systemic pharmacokinetics. The third is mechanism validation using the same batch, therapeutic rather than exclusively preventive dosing, appropriate controls, causal perturbation, and tissue- or cell-resolved readouts. Figure 2 presents these steps as sequential translation gates.

The clinical priority is a prospective, multicenter, adequately powered, randomized trial with central randomization, concealed allocation, matching placebo or double-dummy masking where feasible, and intention-to-treat analysis. A mild-to-moderate induction population on stable background mesalazine may provide an ethical add-on design. The primary endpoint could be clinical remission plus endoscopic improvement at week 8 or 12, with histology, fecal calprotectin, quality of life, traditional-pattern scores, pharmacokinetics, and safety as key secondary outcomes. A maintenance phase should assess steroid-free durability, relapse, hospitalization, and cumulative toxicity.

A factorial component trial could answer a different question: whether the complete five-material prescription provides value beyond a principal component or simplified combination. Such a study requires careful safety justification and chemically characterized arms. Adaptive designs may help discontinue uninformative combinations, but should not sacrifice interpretable comparisons. Embedded mechanistic sampling could test whether changes in SCFAs, AhR activity, barrier function, or immune-cell state mediate clinical response. Mediation analysis should be prespecified and temporally ordered, with exposure preceding biomarker change and biomarker change preceding outcome.

The benefit-risk profile cannot be inferred from centuries of use or from short trials alone. *Sophora* alkaloids, Indigo naturalis, and licorice each provide specific safety signals; combinations and enteric delivery may alter exposure. A prospective registry should capture standardized product and lot information, laboratory monitoring, cardiopulmonary symptoms, electrolyte abnormalities, pregnancy exposure, interactions, hospitalization, and outcomes after discontinuation. Pharmacovigilance denominators are essential: case reports identify hazards but cannot estimate incidence, while spontaneous-report counts without exposure data can mislead.

Risk minimization should be tested rather than merely recommended. Examples include marker-based alkaloid limits, restricted course duration, scheduled liver chemistry and potassium testing, warning cards for dyspnea or severe abdominal pain, electronic checks for duplicate licorice or Kushen products, and contraindication criteria. If mucosal benefit can be achieved with low systemic exposure, the colon-targeted design may improve safety; if coating failure or inflammation increases absorption, it may do the opposite. Release and pharmacokinetic data are thus safety data.

### Evidence strengths, limitations, and reporting standards

Strengths include a defined five-material product, a traditional indication that can be prospectively recorded, randomized clinical precedents, complete-formulation preclinical studies, and mechanistic convergence across microbial, immune, epithelial, and inflammatory domains. The existence of an enteric dosage form creates a concrete pharmaceutical hypothesis. Clinically studied Indigo naturalis and increasingly detailed component research strengthen plausibility. These features justify continued investigation and distinguish FSEC from poorly defined mixtures supported only by database predictions.

Limitations are substantial. Product identity and chemical characterization are inconsistent; equivalent English names are not always proven to represent identical batches; many experiments use acute DSS colitis and small samples; pathway studies often lack causal perturbation; dose translation is uncertain; clinical trials are short and frequently use locally defined outcomes; meta-analyses inherit small-study and reporting bias; recent adjunctive data are observational; and long-term, pregnancy, interaction, and rare-event evidence is sparse. This Review is narrative and cannot exclude missed studies or provide a certainty grade. These limitations define the research agenda and the restraint needed in clinical claims.

### Clinical positioning and priority questions

For clinicians, FSEC should be understood as a pharmacologically active multi-material product, not a neutral supplement. Use should not delay escalation for severe disease, and symptom improvement should be confirmed with objective inflammation assessment. Exact product and co-medications matter. For researchers, the highest-yield advance is not another broad network containing hundreds of predicted targets, but a reproducible batch-to-exposure-to-mechanism-to-outcome chain. For reviewers and editors, product-level claims should be checked against the experimental unit, and safety discussion should reflect all five components.

The clinically relevant question is not whether FSEC is ‘effective for UC’ in the abstract, but for whom, at what disease stage, in which therapeutic role, for how long, and against which alternative. A patient with newly diagnosed mild distal disease, a patient with steroid-dependent extensive colitis, and a hospitalized patient with severe inflammation face very different risks. Emerging UC treatment reviews emphasize matching mechanism and safety to disease biology and clinical trajectory rather than using one average effect estimate (Wangchuk et al., 2024). FSEC studies should therefore report extent, severity, prior treatment failure, steroid exposure, hospitalization history, extraintestinal manifestations, infection screening, and baseline objective inflammation.

Patient priorities also influence the value of an intervention. Oral administration, cultural familiarity, affordability, fear of immunosuppression, pregnancy planning, convenience, and expectations about ‘natural’ products can affect acceptance and adherence. These factors should be measured with validated preference and quality-of-life instruments rather than assumed. Shared decision-making requires balanced communication: the product has preliminary efficacy signals and identifiable pharmacology, but long-term comparative effectiveness and rare harms remain uncertain. A preference for traditional medicine does not remove the need for diagnostic confirmation, objective monitoring, rescue criteria, or escalation when treatment targets are not reached.

Treatment sequencing should be evaluated explicitly. An add-on trial after inadequate response to optimized 5-ASA answers whether FSEC provides incremental benefit; a head-to-head induction trial answers a different question; and a maintenance-withdrawal design tests durability. Studies that combine these scenarios obscure interpretation. Concomitant corticosteroid tapering, mesalazine dose, diet, probiotics, antibiotics, and other Chinese medicines should be standardized or carefully recorded. Pragmatic trials can later assess effectiveness in routine care, but explanatory trials are first needed to establish whether the characterized product itself changes outcomes.

A future article should allow a reader to trace every major claim to an evidence unit. The title and abstract should name the complete product without attributing ingredient-only findings to it. The methods should describe component identities, medicinal parts, processing, extraction or preparation procedures, excipients, dose basis, storage, and analytical characterization. Chemical figures should indicate which compounds were actually measured rather than presenting a decorative catalogue. Mechanism sections should state whether the experiment used the full capsule, an extract, a component, or a purified compound. This reporting discipline would substantially reduce false convergence across the literature.

Preclinical reporting should include species, sex, age, housing, diet, randomization, blinding, sample-size rationale, exclusions, model induction, therapeutic timing, comparator, dose conversion, vehicle, and all tested batches. Microbiome studies need negative controls, sequencing and processing details, absolute or compositional analysis appropriate to the question, and public deposition of raw reads and code. Omics-based target selection should be separated from confirmatory testing. Histology should be scored by masked assessors, and representative images should not substitute for quantified results. Replication by an independent group using the same reference batch would materially increase confidence.

Clinical reporting should follow the relevant randomized-trial or observational-study guideline and include a protocol, registration, statistical analysis plan, participant flow, reasons for missing data, exact intervention accountability, adherence, rescue therapy, and harms by treatment arm. Chinese and English product names should appear together with a stable identifier when available. Primary and secondary outcomes should be labeled consistently across registry, protocol, manuscript, and supplementary files. Null and adverse findings are particularly important because a literature dominated by positive small trials will systematically overestimate benefit.

Five questions should take priority over additional descriptive pathway screens. First, can a reproducible chemical and dissolution specification define FSEC across batches? Second, where and in what form do key constituents and metabolites appear after enteric release? Third, which product-level pathway is causal at a clinically relevant exposure? Fourth, does the characterized capsule improve a validated induction or maintenance endpoint in a rigorous multicenter trial? Fifth, what is the incidence and exposure relationship of hepatic, cardiopulmonary, neurologic, electrolyte, and interaction-related adverse events? Answering these questions would convert a broad plausible network into a testable benefit-risk profile.

Negative answers would also be informative. Failure to demonstrate colonic release would redirect formulation work; absence of exposure-response would challenge a selected marker; loss of benefit after rigorous masking could reveal bias in earlier studies; and a safety signal might identify a narrower dose or population. Ethnopharmacological research is strengthened, not diminished, when traditional knowledge is subjected to falsifiable testing. The appropriate standard is not that a multi-component product must behave like a single synthetic molecule, but that its identity, variability, exposure, effects, and uncertainty are described well enough to support reproducible inference.

## Conclusion

FSEC has a coherent ethnopharmacological rationale and a growing, but not yet definitive, evidence base for UC. Complete-formulation studies support linked effects on microbial metabolites, mucosal immunity, inflammatory signaling, epithelial integrity, and ferroptosis-related biology, while older randomized trials and meta-analyses suggest short-term clinical benefit. Ingredient studies strengthen plausibility and reveal important hazards but cannot substitute for evidence on the defined capsule. The next decisive step is a chemically and pharmaceutically characterized product tested in prospective multicenter trials with contemporary clinical, endoscopic, histologic, biomarker, pharmacokinetic, and safety endpoints. Until then, conclusions should remain provisional and use should be monitored, product-specific, and integrated with standard UC care.
